# Development of a risk scoring system for patients with papillary thyroid cancer

**DOI:** 10.1111/jcmm.14208

**Published:** 2019-02-07

**Authors:** Kyoungjune Pak, Yun Hak Kim, Sunghwan Suh, Tae Sik Goh, Dae Cheon Jeong, Seong Jang Kim, In Joo Kim, Myoung‐Eun Han, Sae‐Ock Oh

**Affiliations:** ^1^ Department of Nuclear Medicine and Biomedical Research Institute Pusan National University Hospital Busan Republic of Korea; ^2^ Department of Anatomy and Department of Biomedical Informatics, School of Medicine Pusan National University Yangsan Republic of Korea; ^3^ Biomedical Research Institute Pusan National University Hospital Busan Republic of Korea; ^4^ Department of Internal Medicine Dong‐A University College of Medicine Busan Republic of Korea; ^5^ Department of Orthopaedic Surgery and Biomedical Research Institute Pusan National University Hospital Busan Republic of Korea; ^6^ Deloitte Analytics Group Deloitte Consulting LLC Republic of Korea; ^7^ Department of Nuclear Medicine and Research Institute for Convergence of Biomedical Science and Technology Pusan National University Yangsan Hospital Yangsan Republic of Korea; ^8^ Department of Anatomy, School of Medicine Pusan National University Yangsan Republic of Korea

**Keywords:** network‐regularized high dimensional cox regression, papillary thyroid cancer, pathway databases, prognosis, TCGA

## Abstract

As the importance of personalized therapeutics in aggressive papillary thyroid cancer (PTC) increases, accurate risk stratification is required. To develop a novel prognostic scoring system for patients with PTC (n = 455), we used mRNA expression and clinical data from The Cancer Genome Atlas. We performed variable selection using Network‐Regularized high‐dimensional Cox‐regression with gene network from pathway databases. The risk score was calculated using a linear combination of regression coefficients and mRNA expressions. The risk score and clinical variables were assessed by several survival analyses. The risk score showed high discriminatory power for the prediction of event‐free survival as well as the presence of metastasis. In multivariate analysis, the risk score and presence of metastasis were significant risk factors among the clinical variables that were examined together. In the current study, we developed a risk scoring system that will help to identify suitable therapeutic options for PTC.

## 1 INTRODUCTION

Thyroid cancer is the most commonly diagnosed cancer in Korea,^1^ and its incidence continues to rise worldwide.^2^ Current treatment options for papillary thyroid cancer (PTC) include surgery, radioactive iodine ablation, and thyroid hormone replacement.^3^ Although thyroid cancer is associated with a generally favorable prognosis, a minority of patients with thyroid cancer experience recurrence or distant metastasis.^3^ Therefore, the challenge remains to distinguish between patients with indolent or aggressive thyroid cancer. An accurate risk stratification of thyroid cancer is essential in order to select the most suitable treatment options. In 2015, the American Thyroid Association proposed a model including low‐, intermediate‐, high‐risk groups for differentiated thyroid cancer (DTC) mostly based on histologic report after surgery.^3^

However, understanding of the molecular basis of pathogenesis and progression in thyroid cancer has progressed.^4^ Genetic mutations in genes such as BRAF, and RAS are associated with both the pathogenesis of DTC and prognosis of thyroid cancer.^5^ In the era of precision medicine, personalized treatment according to potential prognosis for individuals with thyroid cancer is critically important.

Big data have been mass produced for customized diagnosis; however, many medical scientists still use traditional statistical methods such as univariate Cox analysis (1972), the least absolute shrinkage selection operator (Lasso, 1997) and Elastic Net (2005) regression to predict survival.^6^, ^7^ Although these methods have been widely used in survival analysis, they do not incorporate the most up‐to‐date information regarding the complex interplay between biological pathways. A novel variable selection method, so‐called Network‐Regularized high‐dimensional Cox‐regression, has been developed that takes into account signalling pathways and gene networks with the addition of an optional gene‐gene correlation matrix.^7^, ^13^

A new strategy that uses individual information is crucial to accurately stratify patients with PTC. Therefore, we aimed to develop a novel risk scoring system for PTC based on gene networks using The Cancer Genome Atlas (TCGA).

## 2 METHODS

### 2.1 Data acquisition and characteristics

The primary and processed data were downloaded from the Genomic Data Commons Data Portal (https://gdc-portal.nci.nih.gov/) in January 2017. All TCGA data were available without restrictions in publications or presentations according to TCGA publication guidelines. We downloaded mRNA expression data, and clinical information, which was last updated lastly in May 2016. Of 509 cases, the following samples were excluded; metastatic tissues (n = 8); history of other malignancies (n = 33); history of neoadjuvant therapy (n = 4); missing data (n = 9). In total, 455 patients were included in this study.

### 2.2 Selection of genes and risk score

**Figure 1:**
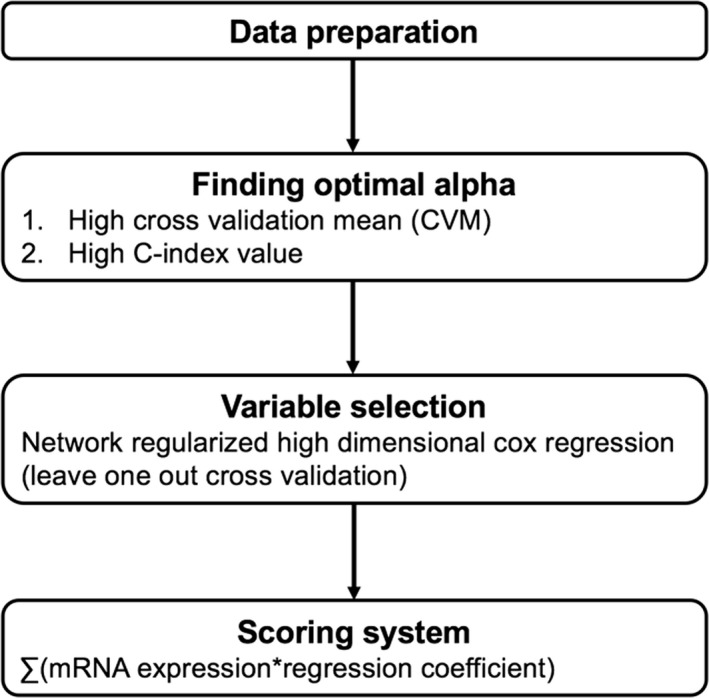
Study protocol

We performed Network‐regularized high‐dimensional Cox regression (Net) using the R package *coxnet *(*version 0.2*) to evaluate the association between event‐free survival (EFS) and mRNA expression. The terms ‘events’ was used to refer to recurrence and/or progression. To obtain more significant results, optional parameters were required. We made a gene‐gene pathway matrix using six large databases (Biocarta, HumanCyc, KEGG, NCI, Panther, and Reactome) as a regularized parameter ‘Ω’ using the R package *graphite*. The mixing parameter α, which decides the balance between Lasso and Ridge, was determined with minimal cross‐validation error. The gene set was selected using Net and the ‘leave‐one‐out cross‐validation (LOOCV)’ method. LOOCV is the most exhaustive cross‐validation methods which train and test on all possible ways to divide the observation into a training and a validation set. Risk score was calculated as the level of expression of each gene, multiplied by the corresponding regression coefficients, consisting of 35 genes in total (Table 1). The cutoff (−5.769287) was determined with maximal Uno's c‐index.^14^ Lower risk scores indicated lower risk for recurrence/progression. The study protocol is presented in Figure 1.

**Table 1:** Selection of genes and regression coefficients for risk score

Variables	Regression coefficients
ADRA2B	−0.07606
ADGRB2	−0.05483
BHMT2	−0.03582
CCBL2	−0.40997
FAM69A	−0.0207
FDXACB1	−0.1655
FTSJ1	−0.06527
IGFBP7	−0.01554
LIMK2	−0.09033
LOC644172	−0.09062
PITRM1	−0.20857
PRMT6	−0.62461
RNF5P1	−0.15293
RPL23AP7	−0.17061
SCARF2	−0.11515
SOCS2	−0.00483
TMEM47	−0.06395
TRIM13	−0.02674
TSC22D3	−0.02379
TSPAN13	−0.06588
TSPAN9	−0.07819
WFDC1	−0.03168
B3GLCT	0.09959
BRAP	0.047107
BUB1	0.043702
SAPCD2	0.051446
CDC20	0.035296
CHAF1B	0.117852
HIST2H2BF	0.07194
KHNYN	0.231944
KIAA1191	0.02546
LANCL2	0.859539
RIBC2	0.048252
TTK	0.103131
ZWINT	0.056916

### 2.3 Statistical analysis

Survival analysis was performed to predict EFS. Variables such as age, sex, histologic subtype, extrathyroidal extension (ETE), lymph node metastasis, distant metastasis, and risk score were assessed using Cox proportional hazard regression analysis. Variables with a p value less than 0.05 were selected for multivariate analysis. To evaluate discriminatory accuracy, we used the *survival* and *survAUC*: log‐rank test, Uno's c‐index for the time‐dependent area under the curve (AUC) and AUC value for t‐year. Survival variables with a c‐index of 0.75 or more were considered to have excellent predictive value for the continuous event time. An AUC value of 0.6 or more was considered acceptable for prediction of t‐year survival. Correlation analysis between risk score and clinical variables was performed by Pearson's Chi‐squared test Yates’ continuity correction because Yates' continuity correction is used in 2 × 2 contingency table when at least one cell of the table has an expected frequency smaller than 5. Statistical analysis was performed using R software version 3.5.0 (The R Foundation for Statistical Computing, 2018), GraphPad Prism 7 for Mac OS X (GraphPad Software Inc, San Diego, CA, USA), and MedCalc software package (ver. 12.6.0.0, MedCalc, Mariakerke, Belgium).

## 3 RESULTS

In total, 455 patients with PTC were included in this study (120 men, 335 women). The mean age was 45.8 years. Of 455 patients with PTC, 43 (9.5%) experienced recurrence/progression during follow‐up (37.0 ± 30.6 months). Patients’ characteristics are summarized in Table 2.

**Table 2:** Patients’ baseline characteristics (n = 455)

Variables	No.
Age (years)	45.8 ± 15.1
Sex	
Male	120
Female	335
Histology	
Papillary thyroid cancer	
Classical	323
Follicular	96
Tall cell	30
Etc	6

### 3.1 Risk scoring system

**Figure 2:**
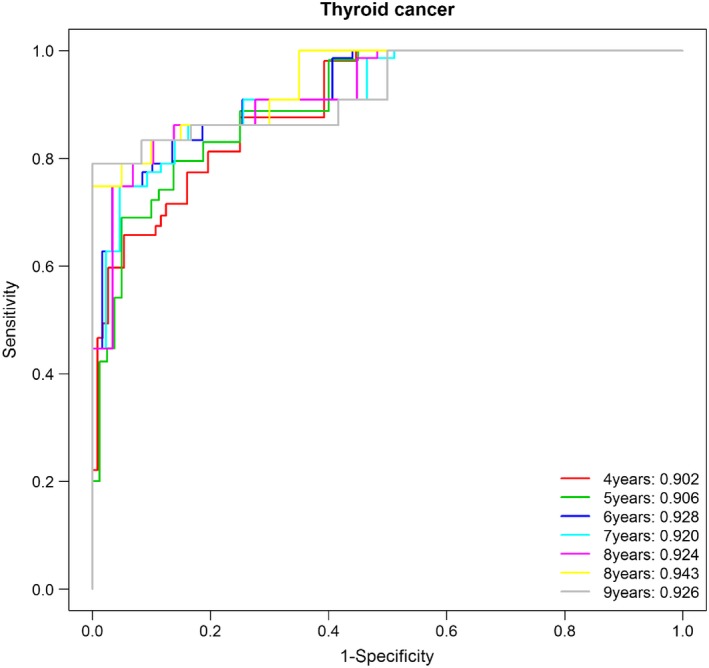
Time‐dependent receiver operating characteristic (ROC) analysis at indicated years

**Figure 3:**
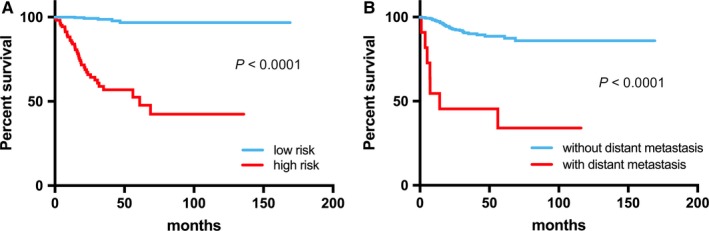
Kaplan‐Meier estimates of event‐free survival (EFS) om papillary thyroid cancer patients according to risk score (A), and distant metastasis (B)

We developed a risk scoring system to predict recurrence/progression of PTC. The risk score ranged between −8.841 and −3.425 for all patients. Time‐dependent receiver operating characteristic analysis showed an acceptable predictive AUC of between 0.902 and 0.943 (Figure 2). A c‐index for the whole course of time was excellent with a value of 0.910. As shown in Figure 3, the risk score was statistically significance (*P* < 0.0001) for EFS as well as the presence of distant metastasis (*P* < 0.0001).

### 3.2 Statistical analysis

**Figure 4:**
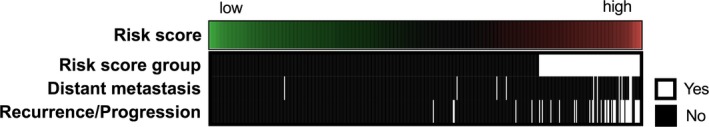
Heatmap of risk score, distant metastasis, and recurrence/progression

Age (≧55 years), sex (male), histologic subtype (classical), ETE (present), lymph node metastasis (present), distant metastasis (present), and risk score (high) were analyzed in relation to EFS by univariate analysis. Older age (hazard ratio 2.0264, 95% confidence interval 1.1083‐3.7051, *P* = 0.0218), presence of ETE (2.0484, 1.1232‐3.7356, 0.0193), presence of distant metastasis (9.8551, 4.3503‐22.3256, <0.0001), and high risk score (32.7945, 12.8901‐83.4343, <0.0001) were significant predictors for EFS, and presence of lymph node metastasis showed a trend towards reduced EFS (1.7761, 0.9689‐3.2555, 0.0632). In multivariate analysis, the presence of distant metastasis (7.2418, 3.1351‐16.7279, <0.0001), and higher risk score (30.7052, 11.9086‐79.1705, <0.0001) were independent predictors for EFS (Figures 3 and 4, Table 3). To identify the correlation between risk score and clinical variables, we performed Pearson's Chi‐squared test Yates’ continuity correction (Table 4). Risk score was highly correlated with age (*P* = 0.009), Stage (*P* = 0.002), M stage (*P* = 0.005), N stage (*P* = 0.003) and ETE (*P* = 0.025) (Table 4).

**Table 3:** Univariate and multivariate analysis of event‐free survival (EFS)

Variables	Univariate			Multivariate		
	Hazard ratio	95% CI	*P* value	Hazard ratio	95% CI	*P* value
Age (≧55 years)	2.0264	1.1083‐3.7051	0.0218			
Sex (male)	1.4431	0.7620‐2.7332	0.2603			
Extrathyroidal extension (present)	2.0484	1.1232‐3.7356	0.0193			
Histologic subtype (classical)	1.0767	0.5524‐2.0985	0.8282			
Lymph node metastasis (present)	1.7761	0.9689‐3.2555	0.0632			
Distant metastasis (present)	9.8551	4.3503‐22.3256	<0.0001	7.2418	3.1351‐16.7279	<0.0001
Risk score (high)	32.7945	12.8901‐83.4343	<0.0001	30.7052	11.9086‐79.1705	<0.0001

**Table 4:** Associations between the risk score and clinical variables

Variables	Risk group		*P* value
	Low risk (n = 348)	High risk (n = 107)	
Age (years)			
<55	256 (73.6)	64 (59.8)	0.009
≥55	92 (26.4)	43 (40.2)	
Sex			
Female	256 (73.6)	79 (73.8)	1
Male	92 (26.4)	28 (26.2)	
Stage			
I & II	342 (98.3)	98 (91.6)	0.002
III & IV	6 (1.7)	9 (8.4)	
M stage			
M0	344 (98.9)	100 (93.5)	0.005
M1	4 (1.1)	7 (6.5)	
N stage			
N0	209 (60.1)	46 (43.0)	0.003
N1	139 (39.9)	61 (57.0)	
Extrathyroidal extension			
None	259 (74.4)	67 (62.6)	0.025
Resent	89 (25.6)	40 (37.4)	

## 4 DISCUSSION

In this study, a risk scoring system derived from mRNA expression values and the presence of distant metastasis were strong predictors for recurrence/progression in patients with PTC. The incidence of thyroid cancer continues to rise worldwide, including in Korea.^2^, ^15^ However, the survival rates for thyroid cancer are relatively good with a 5‐year rate of 97.3%.^16^ Conventional treatment of PTC involves a three‐tiered approach including surgery, radioactive iodine ablation, and replacement of exogenous thyroid hormone, which has remained unchanged since the 1950s.^3^, ^17^ In addition, several reports suggested only active surveillance of low‐risk PTC without surgery.^18^ In this regard, there is a need for new prognostic factors that predict recurrence/progression in thyroid cancer.

The study of cancer genomics have accelerated the convergence of discovery science and clinical medicine.^19^ The molecular characterization of thyroid cancer has begun to influence diagnosis and the overall treatment landscape. Genetic mutations such as BRAF, RAS, and RET are known to be prognostic markers in thyroid cancer.^5^ PTCs with BRAF mutations show a higher risk of recurrence and a higher risk of death.^5^ TERT mutation is also associated with aggressive clinicopathological characteristics and poorer prognosis in PTC.^20^ TCGA launched in 2013 with 33 different tumor types from 11 000 patients.^21^ Data from 507 patients with PTC were included in TCGA generated by raw sequencing, transcriptome profiling, simple nucleotide variation, and copy number variation.^21^ Using the data from TCGA, upregulation of SLC2A1, SLC2A3 and SLC2A14 were associated with increased risk of death in PTC patients,^22^ and downregulation of long non‐coding RNA271 was associated with increased recurrence.^23^ Previous studies have shown the prognostic value of specific genes for other cancers via traditional statistical methods such as Cox regression analysis, Lasso and Elastic net regression.^7^, ^9^, ^10^ These methods have several limitations; Cox regression analysis does not consider gene‐gene expression networks and biological pathways. Lasso and Elastic net regression do incorporate gene‐gene expression correlation by grouping variable selection methods; however, they do not consider biological pathways.^7^, ^13^ Therefore, genes with significant prognostic value as identified by in traditional approaches may have the weakness of overfitting, however, fit in their own dataset. In this study, to overcome these limitations, we developed a novel risk scoring system using Network‐Regularized high‐dimensional Cox regression analysis that incorporated biological pathways as a regularized parameter. To obtain more biological information, we constructed a gene‐gene pathway matrix using six largest pathway databases (Biocarta, HumanCyc, KEGG, NCI, Panther, and Reactome).

In this study, a total of 35 genes were included in risk score system. As lower risk scores indicate the favorable prognosis, the higher expression of 22 genes with a negative regression coefficient, and the lower expression of 13 with a positive regression coefficient corresponds to less recurrence/progression. Among 22 genes with a negative regression coefficient, loss of IGFBP7 expression showed a role in thyroid carcinogenesis,^24^ LIMK2 showed a potential role as a tumor suppressor,^24^ consistent with this study. However, SOCS‐2 proteins showed roles in development and pathogenesis of PTC,^25^ and higher TSPAN13 expression was associated with poor prognosis in a previous report by Li et al.^26^ Among 13 genes with a positive regression coefficient, higher BUB1 expressions were associated with aggressive nature,^27^ consistent with this study.

The mixing parameter α, which decides the balance between Lasso and Ridge, was determined with minimal cross‐validation error. The gene set was selected using Net and the ‘leave‐one‐out’ method for cross‐validation. Risk score was calculated as the level of expression of each gene, multiplied by the corresponding regression coefficients, consisting of 35 genes in total (Table 1). The cutoff (−5.769287) was determined with maximal Uno's c‐index.^14^ Lower risk scores indicated lower risk for recurrence/progression. The study protocol is presented in Figure 1.

In addition to a risk scoring system derived from mRNA, the presence of distant metastasis was an independent predictor of recurrence/progression in this study, which is consistent with a previous meta‐analysis.^28^ Sabet et al suggested that distinguishing synchronous from metachronous manifestation of distant metastases adds an important prognostic feature to risk stratification in DTC.^29^ However, as less than 5% of patients with PTC present with distant metastasis, and imaging modalities such as ^18^F‐Fluorodeoxyglucose positron emission tomography are not routinely recommended, the presence of distant metastasis may be scarcely detected.^3^, ^30^ Therefore, risk stratification according to the presence or absence of distant metastasis may be beneficial for a minority of patients with PTC. In this study, cutoff value of 55 years was used, which has been newly adopted in the American Joint Committee on Cancer (AJCC) staging system (8th edition).^3^ A recent analysis concluded that increasing the age from 45 to 55 years would help avoid overtreatment in 12% of patients, while improving the statistical validity of the model.^31^ Older age was associated with recurrence/progression in PTC with a hazard ratio of 2.0264. In addition, ETE of PTC predicted recurrence/progression in univariate analysis, but not multivariate analysis. Previous studies showed conflicting results regarding the prognostic value of ETE. According to a meta‐analysis by De‐Tao et al, minimal ETE is a risk factor for recurrence.^32^ However, it was excluded from the parameters for DTC in the AJCC 8th edition,^3^ and was considered useful for T3 staging in the AJCC 7th edition.^33^ In this study, 25.7% of PTCs had minimal ETE and 2.6% had macroscopic ETE, however, was not an independent predictor of recurrence/progression. There were several limitations to this study. All data were retrospectively collected and derived from TCGA. In addition, as a small number of patients died during follow‐up, we could not validate the risk scoring system with overall survival, or disease‐specific survival.

As the importance of personalized therapeutics in aggressive PTC increases, an accurate risk stratification system is increasingly required. In the current study, we developed a novel risk scoring system for PTC derived from mRNA expression values, which is an independent predictor of prognosis in PTC. Although some limitations exist, our results provide insight into the prognostic prediction of patients with PTC.

## CONFLICT OF INTEREST

The authors declare that they have no conflict of interest.
